# Jack Straws

**Published:** 1916-03

**Authors:** 


### Jack Straws.

We need not describe this good old fashioned game. We simply want to emphasize that it ought to be part of the armamentarium of every child as a training of muscular co-ordination and the muscular sense. It should also be used in cases in which, from paralysis or other cause, it is necessary to retrain these faculties. Incidentally, it might prove an alternative for diversion of convalescents, and might possibly distract the attention in such a way as to ameliorate some of the manifestations of mental disease.

